# Gene recoding by synonymous mutations creates promiscuous intragenic transcription initiation in mycobacteria

**DOI:** 10.1128/mbio.00841-23

**Published:** 2023-10-03

**Authors:** Nuri K. Hegelmeyer, Lia A. Parkin, Mary L. Previti, Joshua Andrade, Raditya Utama, Richard J. Sejour, Justin Gardin, Stephanie Muller, Steven Ketchum, Alisa Yurovsky, Bruce Futcher, Sara Goodwin, Beatrix Ueberheide, Jessica C. Seeliger

**Affiliations:** 1 Department of Pharmacological Sciences, Stony Brook University, Stony Brook, New York, USA; 2 Department of Microbiology and Immunology, Stony Brook University, Stony Brook, New York, USA; 3 Proteomics Laboratory, New York University Grossman School of Medicine, New York, New York, USA; 4 Cold Spring Harbor Laboratory, Cold Spring Harbor, New York, USA; 5 Department of Biochemistry and Molecular Pharmacology, New York University Grossman School of Medicine, New York, New York, USA; Weill Cornell Medicine, New York, New York, USA

**Keywords:** recoding, codon pair bias, transcription, *Mycobacterium*, tuberculosis, translation

## Abstract

Each genome encodes some codons more frequently than their synonyms (codon usage bias), but codons are also arranged more frequently into specific pairs (codon pair bias). Recoding viral genomes and yeast or bacterial genes with non-optimal codon pairs has been shown to decrease gene expression. Gene expression is thus importantly regulated not only by the use of particular codons but also by their proper juxtaposition. We therefore hypothesized that non-optimal codon pairing could likewise attenuate *Mycobacterium tuberculosis* (*Mtb*) genes. We explored the role of codon pair bias by recoding *Mtb* genes (*rpoB, mmpL3*, and *ndh*) and assessing their expression in the closely related and tractable model organism *M. smegmatis*. To our surprise, recoding caused the expression of multiple smaller protein isoforms from all three genes. We confirmed that these smaller proteins were not due to protein degradation but instead issued from new transcription initiation sites positioned within the open reading frame. New transcripts gave rise to intragenic translation initiation sites, which, in turn, led to the expression of smaller proteins. We next identified the nucleotide changes associated with these new sites of transcription and translation. Our results demonstrated that apparently benign, synonymous changes can drastically alter gene expression in mycobacteria. More generally, our work expands our understanding of the codon-level parameters that control translation and transcription initiation.

**IMPORTANCE:**

*Mycobacterium tuberculosis* (*Mtb*) is the causative agent of tuberculosis, one of the deadliest infectious diseases worldwide. Previous studies have established that synonymous recoding to introduce rare codon pairings can attenuate viral pathogens. We hypothesized that non-optimal codon pairing could be an effective strategy for attenuating gene expression to create a live vaccine for *Mtb*. We instead discovered that these synonymous changes enabled the transcription of functional mRNA that initiated in the middle of the open reading frame and from which many smaller protein products were expressed. To our knowledge, this is one of the first reports that synonymous recoding of a gene in any organism can create or induce intragenic transcription start sites.

## INTRODUCTION

Across the tree of life, genomes encode some codons more commonly than others, a preference known as codon usage bias. Independent of this well-known phenomenon, each organism also exhibits a preference for certain codon pairs. First termed 25 years ago, codon *pair* bias (CPB) describes the preferential use of some codon pairs over other synonymous pairs (1). Not long after the first genomes were sequenced, early bioinformatics studies reported on the prevalence of codon pair bias, which was observed in diverse coding genomes, from eukaryotes to bacteria (2 – 4).

As the more extensively studied phenomenon, codon usage bias has been applied broadly across organisms toward optimizing or, less often, attenuating gene expression (5). By contrast, most codon pair bias studies to date have sought to impair gene expression, predominantly in viral pathogens. Prior codon pair bias studies have shown that gene expression can be attenuated by synonymously recoding viral genomes so they include more rare codon pairs without perturbing codon usage in a process called codon pair deoptimization. One of the first reports on the functional consequences of codon pair bias demonstrated that synonymously recoding the poliovirus genome by codon pair deoptimization suppressed viral gene expression, reduced viral replication, and yielded a virus that protected mice against polio when used as a vaccine (6). Subsequent studies investigating gene attenuation by altering codon pairs suggest that the mechanism of attenuation is most likely multifactorial. In yeast, deoptimizing some codon pairs caused changes in translation efficiency (7, 8), while recoding an influenza gene decreased the half-life of the viral mRNA (9). Although the exact mechanism remains elusive, synonymous recoding by codon pair deoptimization has been applied to attenuate several other pathogenic viruses, including respiratory syncytial virus, Zika virus, and the recently emergent coronavirus SARS-CoV-2 (10 – 18).

By contrast, there has been only one study on codon pair deoptimization to attenuate a bacterial pathogen. Coleman et al. sought to attenuate *Streptococcus pneumoniae* by synonymously recoding a single gene, the virulence factor pneumolysin (*ply*) (19). Recoding *ply* led to reduced Ply expression and rendered the mutant less virulent than the wild type. However, the mechanism by which deoptimizing codon pair bias compromises bacterial gene expression has not, to our knowledge, been explored.

*Mycobacterium tuberculosis* (*Mtb*) is the causative pathogen of the ancient and deadly infectious disease tuberculosis (TB). TB caused an estimated 1.3 million deaths in 2021 and remains among the leading infectious causes of death worldwide, second only to COVID-19 (20). The attenuated strain *Mycobacterium bovis bacille* Calmette-Guérin, or BCG, is given worldwide to newborns as a vaccine against severe TB but is not considered protective against pulmonary TB, which is the primary cause of mortality. Based on previous studies with viral pathogens and *S. pneumoniae*, we hypothesized that synonymously recoding essential genes in *Mtb* would attenuate protein expression, thereby reducing bacterial survival and pathogenicity. We chose to recode the codon pair bias in several *Mtb* genes: the RNA polymerase β-subunit *rpoB* (*rv0667*) and the trehalose monomycolate exporter *mmpL3* (*rv0206c*), which are essential in normoxic conditions (21 – 23), and the type-II NADH dehydrogenase *ndh* (*rv1854c*), which is essential under hypoxia and on host-related carbon sources (22, 24). We first sought to confirm that recoding attenuated protein expression but found instead that recoding results in the expression of many smaller protein isoforms that appeared to initiate from recoded portions of the open reading frame (ORF). This unexpected result prompted us to investigate mechanisms of pervasive intragenic expression.

Here, we report the impact of synonymous recoding on translation and transcription initiation in *Mtb* genes. We examined the expression of wild-type (WT) and recoded *Mtb* genes and found that synonymous mutations can give rise to intragenic transcription start sites (TSS) that produce functional mRNA. We further demonstrated that the smaller protein isoforms expressed from transcripts that initiated from *de novo* intragenic promoters. Finally, we uncovered evidence that the coding genome exhibits bias against codon pairs or nucleotide hexamers that can cause transcription, indicating that these higher-order patterns in the genetic code may serve to moderate gene expression. Taken together, our findings reveal that certain synonymous changes can drastically alter the way genes are expressed in mycobacteria, further supporting the growing consensus that synonymous mutations are not always silent.

## RESULTS

### Synonymous mutations to introduce rare codon pairs cause the expression of additional protein products

Previous work on codon pair bias and gene expression in viruses and *S. pneumoniae* (6, 19) led us to hypothesize that synonymous recoding to introduce rare pairs of codons, also known as codon pair deoptimization, would similarly attenuate gene expression in mycobacteria. We further hypothesized that recoding longer gene segments would lead to a higher degree of attenuation.

**Fig 1:**
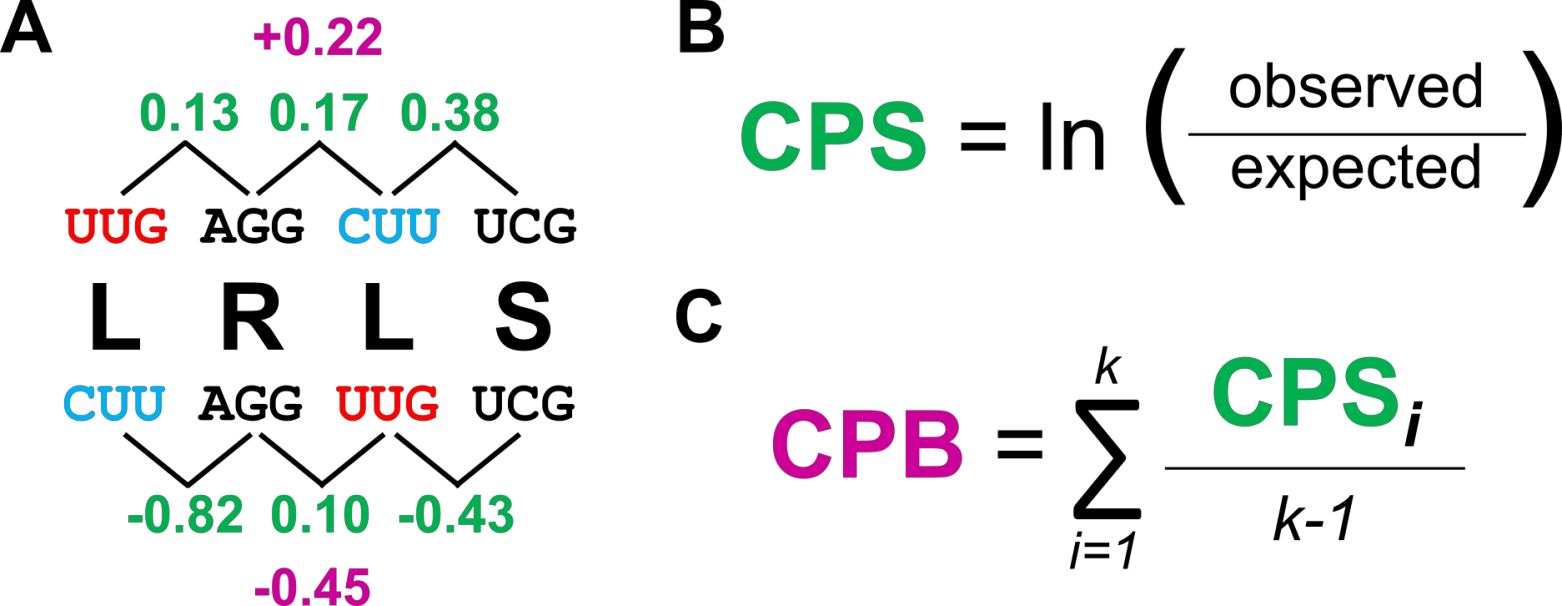
The codon pair bias (CPB) of a coding sequence is scored as the mean of each codon pair score. (**A**) A short coding sequence encoding Leu-Arg-Leu-Ser can be coded in two ways, shown above and below the peptide sequence. Each Leu is encoded by a unique, synonymous codon, UUG or CUU. The positions of these two Leu codons are swapped in the top and bottom sequences, effectively changing the codon pair biases although the codon usage is the same. (**B**) The codon pair score (CPS; green) is determined by calculating the natural log of the ratio of the total number of times a given codon pair is observed in an organism’s coding genome to the number of times the codon pair is expected to appear, controlling for codon usage (see also Methods). (**C**) The CPB score (magenta) assigned to a given gene is the arithmetic mean of all the CPS found in the sequence. Figure adapted from reference (6).

The codon pair bias of a sequence is described using codon pair scores (CPS; Fig. 1A). A codon pair score is derived from the natural log transformation of the ratio of the observed frequency of a given codon pair (i.e., in-frame hexamer) to the expected frequency, controlling for the number of times the corresponding dipeptide is encoded across the entire coding genome (Fig. 1B). Positive scores indicate “enriched” codon pairs, relative to other codon pairs encoding the same dipeptide, while negative scores describe “depleted” pairs. For example, the hexamer TTA-CGT represents the most enriched codon pair for the Leu-Arg dipeptide in *Mtb* and has a codon pair score of 0.724, whereas the least common Leu-Arg dipeptide in *Mtb* is CTC-CGG with a codon pair score of −1.430. A gene can then be described by a codon pair bias score, which is the mean of codon pair scores for all in-frame hexamers (Fig. 1C, see also: Methods). Substituting CTC-CGG for TTA-CGT does not change the amino acid sequence—and is thus a synonymous mutation—but does replace a commonly occurring codon pair with a rarer one, thereby decreasing the codon pair bias score for a gene. Thus, a codon pair bias score indicates how far codon pair bias within a single coding sequence deviates from the coding genome as a whole. However, since codon pair bias score is an average, a gene can contain very rare codon pairs whose effects on the score are offset by commonly occurring codon pairs elsewhere in the sequence; using codon pair bias score as a metric assumes that the effects of codon pair bias on gene expression are aggregate and not individual. As previously mentioned, synonymously rearranging the codons within a gene to increase the number of rare codon pairs is a process known as codon pair deoptimization. Hereafter, we refer to this process as “recoding.”

**Fig 2:**
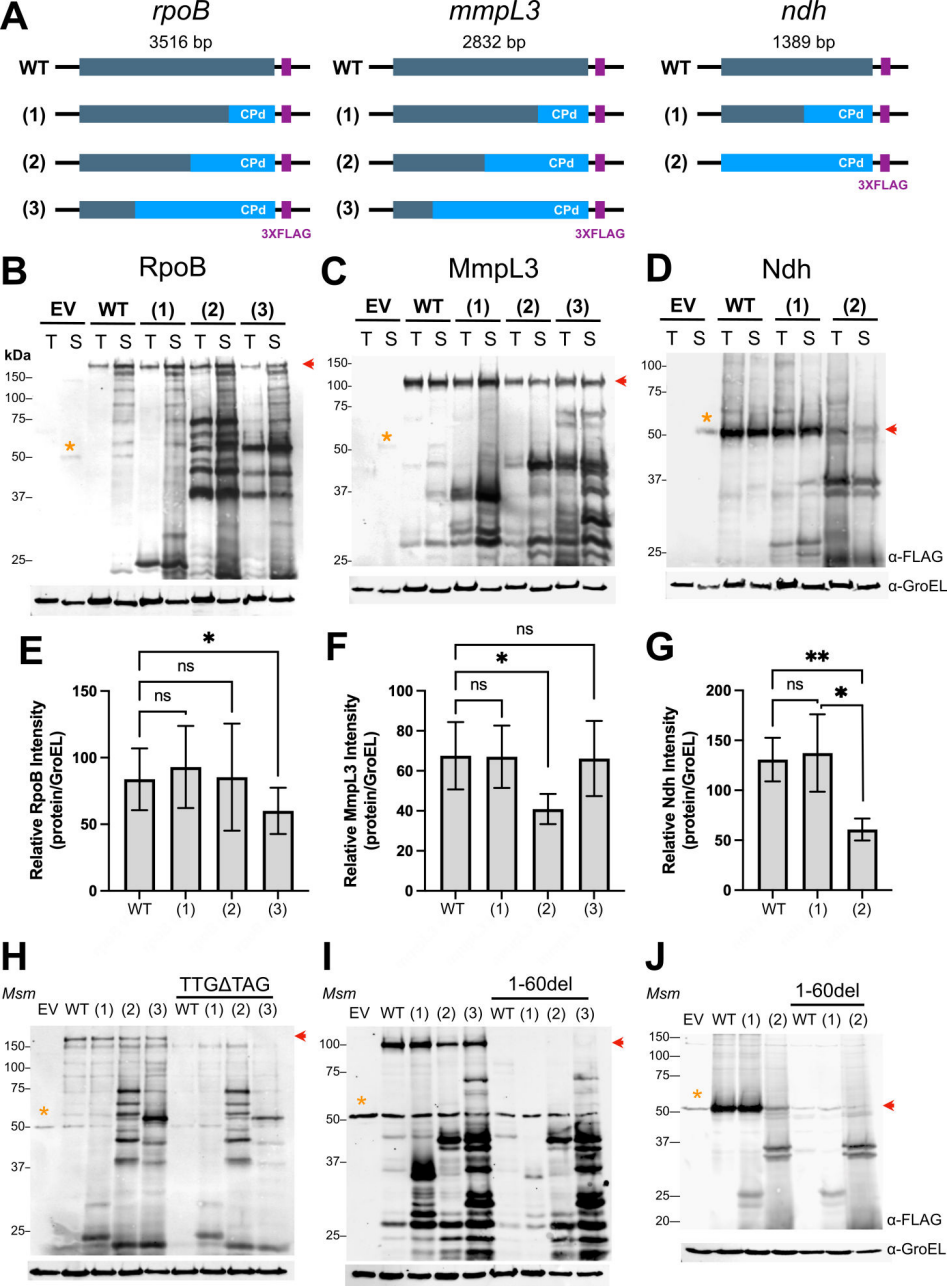
Synonymous mutation by codon pair deoptimization causes expression of smaller peptides in mycobacteria. (**A**) Diagram of the strategy to codon pair deoptimize *Mtb* genes and assess their expression. Different lengths of *rpoB*, *mmpL3*, and *ndh* sequences were recoded starting from the 3′ ends and fused with a 3XFLAG peptide epitope at the C-terminus. Anti-FLAG immunoblots of total lysates from *Mtb* (T) or *Msm* (S) carrying wild-type and recoded *Mtb* genes (**B**) *rpoB*, (**C**) *mmpL3*, or (**D**) *ndh* expressed from a multi-copy episomal plasmid. (**E–G**) Quantification of full-length protein from each *Msm* strain in (**B–D**) normalized to a GroEL loading control. Data represent the average ± S.D. of *n* = 5 independent experiments. Repeat measures one-way ANOVA with Dunnett’s multiple comparison analysis was performed to test significance. *P < 0.05, ***P < 0.002. Anti-FLAG immunoblots of *Msm* strains in which (**H**) the start codon of *rpoB* was replaced with a stop codon (TTGΔTAG) or (**I and J**) the first 60 nt were deleted from *mmpL3* and *ndh*. Immunoblots are representative of ≥3 independent experiments. Red arrows indicate the expected migration of the full-length proteins. Yellow asterisks indicate a non-specific FLAG cross-reacting band in *Msm* total lysates.

In a pilot study, we selected several genes that are required for the survival of *Mtb* under normoxic (RNA polymerase β-subunit *rpoB*; trehalose monomycolate transporter *mmpL3*) or hypoxic (type-II NADH dehydrogenase *ndh*) culture conditions. For this test system, we recoded the genes by shuffling codons within each reading frame to maximize the occurrence of rare codon pairs without significantly altering codon usage. For each gene, we further generated up to three variants in which we recoded different length segments of the ORF starting from the 3′ ends (Fig. 2A). Each variant was independently recoded and so represents a unique sequence, and the codon pair bias score was deoptimized relative to bias in *Mtb* coding genome (see Methods). For consistent expression, each variant encoded a C-terminal 3XFLAG epitope fusion and was expressed from a multicopy episomal plasmid in which transcription was driven by the constitutive *groEL* promoter (P*groEL*) (25). For initial experiments assessing protein expression, these constructs were transformed into the fast-growing, non-pathogenic species *M. smegmatis* (*Msm*).

Contrary to our hypotheses, variants encoding segments with more rare codon pairs did not consistently lead to decreased protein expression (Fig. 2B through G). Instead, a wholly unexpected and striking feature was the detection of multiple peptides at molecular weights lower than that of the corresponding full-length protein (Fig. 2B through D). This phenomenon appeared to be general, as the additional protein products were observed for all recoded variants of all three genes. Also, the additional proteins were not an artifact of expression of *Mtb* genes (i) in the heterologous host *Msm*, (ii) from multiple gene copies, or (iii) under selection with the translational inhibitor hygromycin, as the pattern and relative intensities of the additional proteins were similar when the variants were expressed in *Mtb* or *Msm* (Fig. 2B through D) or from a single stably integrated gene copy and in the absence of hygromycin (Fig. S1). Importantly, these results supported our further use of the experimentally tractable, fast-growing, and non-pathogenic species *Msm* to study the effects of recoding *Mtb* genes. Lastly, the additional proteins appeared to derive directly from the recoded gene segment since the size range of the new products corresponded qualitatively with the length of the recoded segment.

### Smaller proteins express from new translation initiation sites within recoded gene regions

We noted that the additional proteins must be in-frame isoforms since they were detected by anti-FLAG immunoblot and therefore must contain a C-terminal FLAG tag. Furthermore, they are unlikely the product of degradation, since every gene variant encodes the wild-type amino acid sequence and there is no known mechanism by which a protease can discriminate between identical proteins. Nevertheless, to confirm that the smaller isoforms were not degradation products, we pursued two strategies to preclude translation of the full-length protein. First, we substituted the predicted start codon with a stop codon (TTGΔTAG). This mutation abolished the expression of full-length RpoB, but not the smaller isoforms (Fig. 2H). For Ndh and MmpL3, replacing the start codon with the TAG stop codon did not abolish the expression of the full-length Ndh or MmpL3 (Fig. S2). We speculated that apparent full-length protein was still expressed because the actual (or additional) translation initial site(s) (TIS) were located just downstream of the predicted start codon. Thus, for Ndh and MmpL3, we created additional variants in which we deleted the first 60 nucleotides (20 aa) of the ORF, including the start codon (Δ1–60nt). This deletion abolished the expression of full-length Ndh and MmpL3, but not that of the corresponding smaller isoforms (Fig. 2I and J). Overall, these results confirmed that smaller proteins were not the result of post-translational cleavage. We therefore reasoned that they must originate from TIS within the ORF and, more specifically, from within the recoded segments.

### Codon pair bias score of the gene does not determine the size or number of smaller proteins expressed

We observed no clear correlation between the length of the gene recoded and the quantity of smaller proteins expressed as represented by unique bands on immunoblot (Fig. 2B through D). However, the question remained: If the recoded length is controlled, does the codon pair bias score of a recoded gene influence the size and quantity of smaller proteins produced? In other words, does intragenic expression arise from global changes across the gene or to local instances of rare codon pairs? To answer this question, we generated additional codon pair bias score variants, this time recoding the same sequence segment as in *rpoB*(3) (2,394 of 3,516 bp), which had the longest recoded segment and thus the highest theoretical probability of achieving a wide range of codon pair bias scores upon recoding. Starting from the wild-type sequence, we recoded this segment, this time by generating 100,000 randomly shuffled sequences. From this pool, we selected sequences with a range of codon pair bias scores between those of *rpoB*(3) (−0.14) and the wild type (0.06).

**Fig 3:**
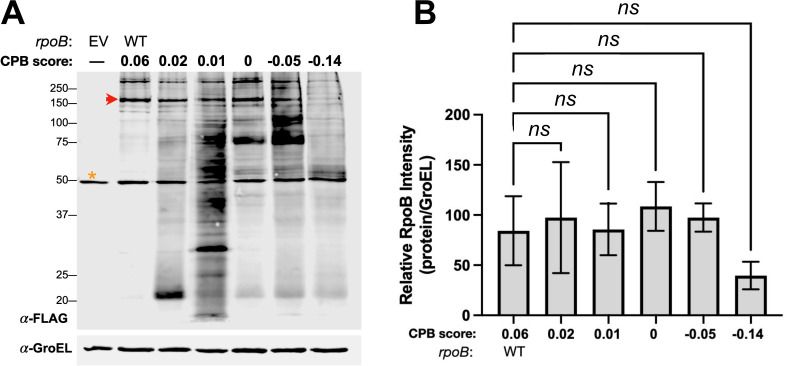
The number of smaller proteins and the expression of full-length proteins vary but do not correlate with the codon pair bias score. (**A**) Anti-FLAG immunoblot of *Msm* strains carrying wild-type *Mtb rpoB* or recoded variants with the indicated codon pair bias scores. The red arrow indicates the expected migration of full-length RpoB. The yellow asterisk indicates a non-specific FLAG cross-reacting band in *Msm* total lysates. Immunoblot is representative of three independent experiments. (**B**) Quantification of full-length RpoB normalized to a GroEL loading control. Repeat measures one-way ANOVA with Dunnett’s multiple comparison analysis was performed to determine significance in *n* = 3 independent experiments. All pairwise comparisons to WT were not significant (ns; *P* > 0.05).

We then assessed protein expression from these sequences to determine whether the levels of smaller protein isoforms could be tuned by codon pair bias score. As was the case when different sequence lengths were recoded, no obvious correlation was observed between the codon pair bias score and expression of full-length RpoB (Fig. 3). Furthermore, there was no clear correlation between codon pair bias score and the number, size, and amounts of additional protein products. These results suggested that the codon pair bias score, which describes the cumulative effect of codon shuffling, does not account for the production of smaller proteins, which we then postulated arises from local and particular sequence changes.

### Synonymous recoding causes the transcription of mRNA that initiates in the middle of the open reading frame and is translated into smaller proteins

**Fig 4:**
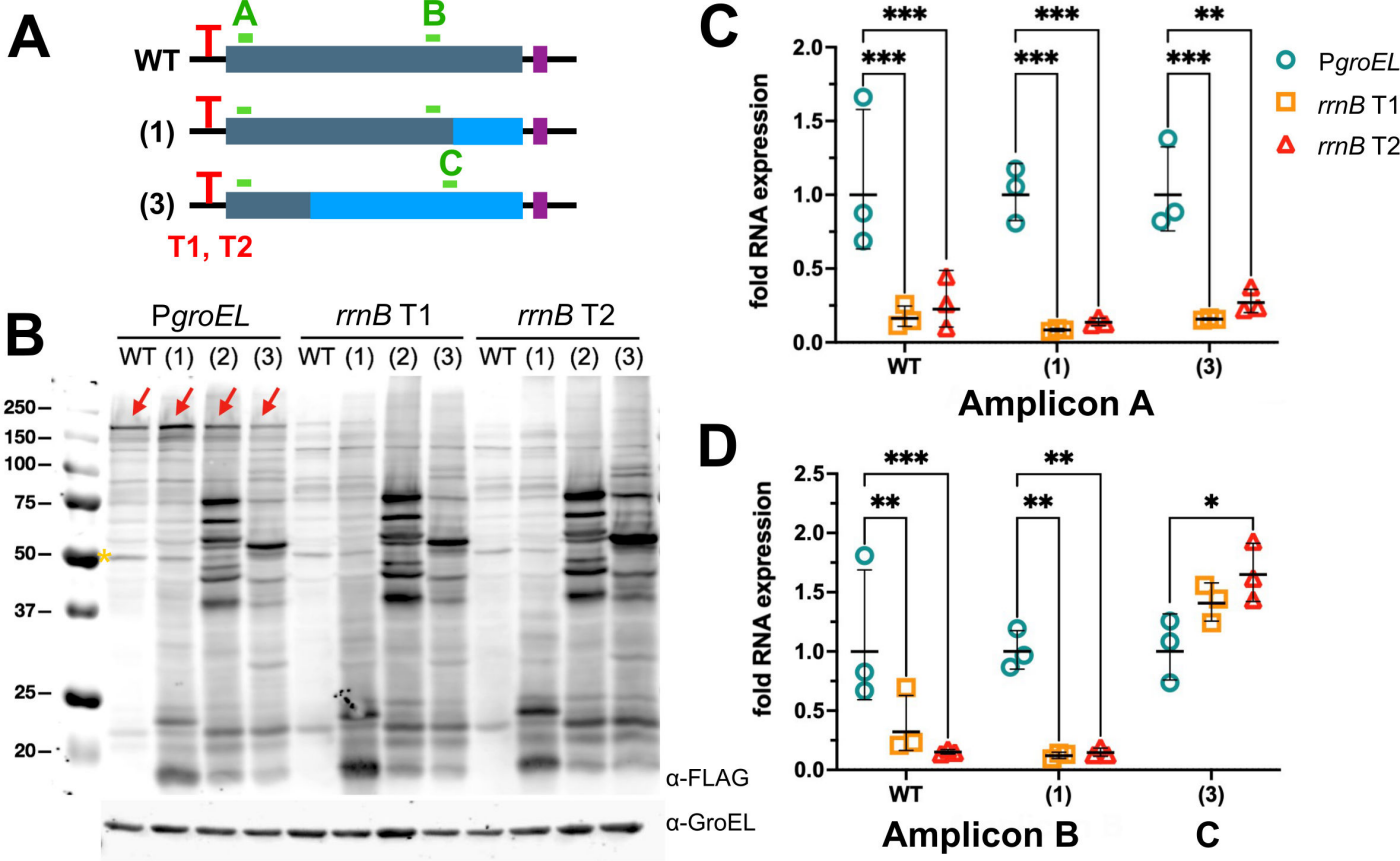
Recoded gene variants express transcripts that initiate from within recoded regions. (**A**) Diagram of *rpoB* genes in which the *groEL* promoter (P*groEL*) was substituted by the *E. coli rrnB* T1 or T2 terminator loop (red “T”) to repress full-length transcript. Amplicon A probes a 5′ segment encoding the wild-type sequence in all variants. Amplicon B probes a 3′ segment that is wild-type in WT and variant (2) and amplicon C probes a segment that is recoded in variant (3). (**B**) Anti-FLAG immunoblot of total lysates from *Msm* strains carrying WT *rpoB* or recoded variants. GroEL was used as a loading control. The yellow asterisk indicates a non-specific FLAG cross-reacting band in *Msm* total lysates. Immunoblot is representative of *n* = 3 independent experiments. (**C and D**) qPCR of amplicons A, B, and C for *Msm* strains carrying WT *rpoB* or recoded variants. All samples were normalized to 16S rRNA. For each amplicon, the average value from each 5′ variant (P*groEL*, *rrnB* T1, and *rrnB* T2) was then normalized to PgroEL. Data and average ± S.D. from *n* = 3 independent experiments are shown. Two-way ANOVA was performed to determine significance (*P < 0.05; **P < 0.02; ***P < 0.002).

Having confirmed that recoding introduces additional TIS in the middle of the ORF, we next asked whether transcriptional changes contribute to these new sites of protein synthesis. We hypothesized that introducing rare codon pairs can result in new TSS. These new, shorter mRNAs can contain new TIS and lead to the expression of smaller proteins. To test this hypothesis, we sought to prevent the production of the full-length transcript by replacing the *groEL* promoter with the *Escherichia coli rrnB* T1 or T2 terminator loops, which function as strong transcriptional terminators in *Msm* (Fig. 4A) (26). Immunoblot analysis showed that, as expected, the expression of the full-length RpoB was reduced or abolished, but smaller isoforms persisted (Fig. 4B). We confirmed that either terminator T1 or T2 strongly inhibited the transcription of full-length *rpoB* mRNA using quantitative PCR of a 5′ amplicon common to all variants and the wild type (Fig. 4C). By contrast, qPCR of appropriate 3′ amplicons revealed that the recoded region of *rpoB*(3) was still transcribed (Fig. 4D). We thus concluded that recoding using synonymous mutations introduced TSS within the ORF.

### 5′-end sequencing confirms transcription initiation within recoded regions

**Fig 5:**
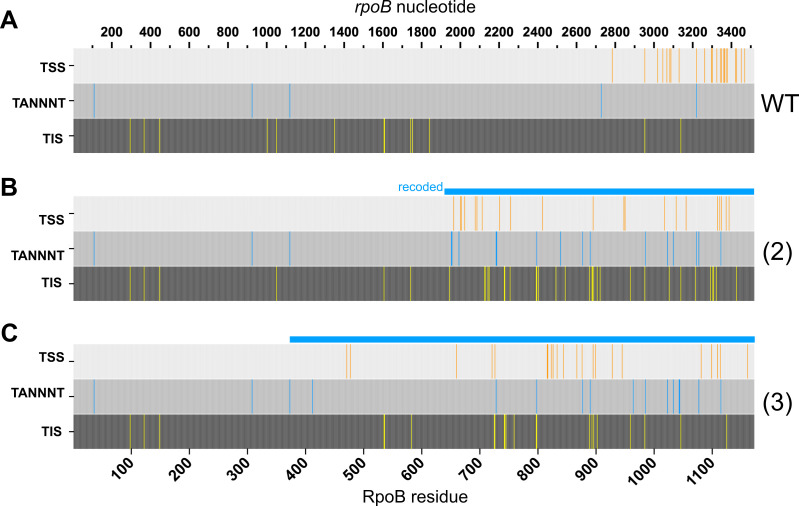
Gene recoding gives rise to a higher incidence of intragenic transcription and translation initiation. TSS identified by 5′ RACE and TIS identified by detection of N-terminally modified peptides for *Msm* strains carrying (**A**) wild-type *rpoB,* (**B**) variant (2), or (**C**) variant (3). The positions of TANNNT transcription initiation motifs are also indicated. Nucleotide position is indicated on the top axis; amino acid position on the bottom axis. The light blue bar above each gene indicates the recoded region. TSS were detected in at least two of three independent experiments. TIS were detected in both of two independent experiments.

What specific sequence changes within recoded regions give rise to intragenic TSS and TIS? To answer this question, we mapped the 5′ ends of mRNA transcripts by 5′ rapid amplification of cDNA ends (RACE), a method that selectively amplifies TSS. We hypothesized that RACE would identify more internal TSS (iTSS) in the recoded genes than in the wild type, as this would corroborate our qPCR data indicating that recoding creates new functional intragenic starts. First, we detected iTSS arising from the wild-type gene—a plausible finding since 5′-end mapping transcriptomics have shown that iTSS occur endogenously in mycobacteria (27 – 29). In addition, and in support of our hypothesis, we found that there were more iTSS in recoded variants—and specifically in the recoded portions of variant genes (Fig. 5). The number of iTSS appeared to correspond with the length of recoded sequence such that more intragenic start sites were mapped to *rpoB*(3) vs *rpoB*(2). Furthermore, iTSS in recoded *rpoB*(3) were farther apart than in *rpoB*(2), consistent with immunoblots showing more smaller proteins expressed from the latter (Fig. 2B).

In summary, we found that portions of the gene containing more rare codon pairs gave rise to more iTSS. This result supports a secondary hypothesis that codon pair bias reflects the depletion of codon pairs that stimulate transcription initiation.

### Codon pair deoptimization increases the number of transcription motifs appearing intragenically

Mycobacterial TSS are typically purines and are preceded by the −10 element for the housekeeping sigma factor, SigA (consensus sequence: TANNNT) (27 – 29). If introducing new motifs such as TANNNT or other −10 motifs is in fact how codon pair deoptimization can lead to intragenic transcription, then it would follow that these motifs should be overrepresented in recoded gene regions. To test this hypothesis, we mapped all instances of TANNNT in any frame and compared the number of times they occurred in codon pair deoptimized vs wild-type genes. We also mapped all instances of a second common start motif, ANNNT. Recoded *rpoB* contained 3 to 4 times more TANNNT motifs (Fig. 5). Of note, *rpoB*(2) contained the most TANNNT motifs, which is consistent with the observation that this variant also expressed the greatest number of smaller protein isoforms (Fig. 2B). In contrast, ANNNT sequences were common throughout wild type and recoded regions, with no corresponding change in frequency relative to intragenic TSS (Table S5). We also examined where TANNNTs occurred in relation to the TSS inferred from 5′-end sequencing (Fig. 5; Table S5). Several TANNNT motifs in recoded regions appeared prior to one or more iTSS, as expected if our hypothesis were true. For example, only *rpoB*(2) contained two new TANNNT motifs overlapping at nucleotide positions 1,951 and 1,956, corresponding to downstream iTSS at 1,963 and 1,969. These observations once again support the hypothesis that recoding the codon pair bias of genes enables new iTSS because specific codon pairs give rise to new TSS motifs.

### Mapping protein N-termini confirms translational initiation sites within recoded regions

To explore the relationship between the TSS introduced by recoding and TIS within the resulting transcripts, we detected and mapped the N-termini of the additional RpoB protein products. In contrast to studies that map TIS by profiling ribosomal occupancy on a nucleotide sequence, we mapped TIS by detecting the resulting peptide and the results are therefore complementary to ribosome footprinting. Proteins generated from wild-type and recoded *rpoB* variants were enriched by anti-FLAG antibody affinity prior to N-terminal labeling by dimethylation and tryptic peptide detection by mass spectrometry. Several considerations were made in identifying N-termini as bona fide TIS (see Materials and Methods for details). For this study, we included N-termini that mapped to plausible start codons such as canonical start codons AUG and GUG, or the rarer start codons UUG and CUG.

We mapped detected TIS to the RpoB sequence. For reasons of data quality, results from *rpoB*(1) were not included in our final analysis, but ample comparisons were available based on *rpoB*(2) and *rpoB*(3). The resulting maps of N-terminal peptides confirmed that more TIS were detected in recoded gene segments than in the corresponding regions of the wild-type sequence (Fig. 5). The relative numbers of detected TIS in recoded regions corroborated the multiple isoforms detected by immunoblot (Fig. 2B). TIS at residues 97, 121, 148, 534, 983, and 1,045 were detected robustly across the wild type, *rpoB*(2), and *rpoB*(3). However, 30 and 19 TIS were found from the recoded variants *rpoB*(2) and *rpoB*(3) vs 13 TIS in the wild type. Of note, the position of the TIS corresponded qualitatively with recoded regions containing dense TSS (Fig. 5). Importantly, the higher number of TIS arising from the recoded regions of variant genes was not due to an increase in the absolute number of start codons, since codon usage was largely preserved during recoding.

What do the detected TIS tell us about the start codons that initiate smaller proteins from recoded genes? First, TIS unique to recoded genes appeared more likely to correspond to less common start codons. In wild-type RpoB, only 23% (3 of 13) TIS initiated at the rarer start codons UUG or CUG (compared to the canonical start codons AUG or GUG). By contrast, a higher proportion of TIS initiated at these rarer codons in *rpoB*(2) (57% or 12 of 21) and *rpoB*(3) (50% or 6 of 12). This trend was not an artifact of codon usage changes, as all genes contained similar numbers of in-frame UUG/CUG codons (80, 79, and 73 in the wild type, *rpoB*(2), and *rpoB*(3)). Thus, among all UUG/CUG codons, the percentage that functioned as start codons was higher in recoded variants (11% and 8% in *rpoB*(2) and *rpoB*(3)) than in the wild type (4%). From this data, we inferred that recoding likely affected gene expression mechanisms upstream of translation initiation—mechanisms that would boost the accessibility of start codons such as UUG/CUG to an initiating ribosome.

If changing the start codon does not as a rule disrupt or induce translation initiation, what other nucleotide alterations drive changes in TIS? Shine-Dalgarno (SD) sequences are generally thought to be major drivers for translation initiation in bacteria. We hypothesized that synonymous changes that introduce or disrupt intragenic SD-like motifs contributed to the observed TIS. We therefore analyzed the gene sequences for AAG or GGA in the 25 nt preceding each detected start codon. However, we could not discern any obvious relationship between TIS and SD-like sequences. On the one hand, SD motifs were not necessary, as we observed TIS with and without such motifs in both wild-type and recoded segments. On the other hand, both wild-type and recoded genes contained in-frame, intragenic ATG, or GTG codons that were preceded by an SD motif but were not detected as a TIS. Unfolded mRNA secondary structures upstream of a start codon have also been reported as important for TIS (30, 31). We thus performed a preliminary analysis comparing the secondary structure of 50 or 20 nucleotides upstream of selected N-termini in recoded regions where TIS were detected vs not detected. There was no obvious correlation between predicted mRNA structure and the presence or absence of an inferred TIS (data not shown). These results indicate that SD motifs and permissive RNA secondary structures are neither necessary nor sufficient for intragenic translation initiation.

**Fig 6:**
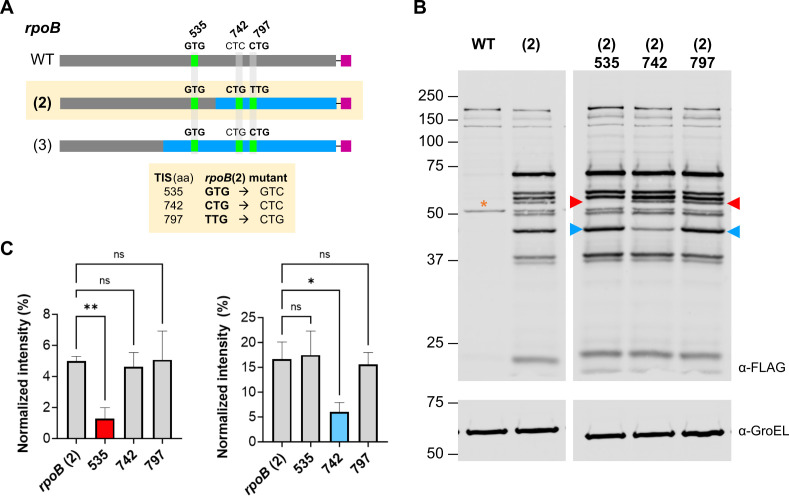
Mutating TIS sites in *rpoB*(2) leads to decreases in the levels of specific protein isoforms. (**A**) Three sites chosen for mutation are annotated with the corresponding codons and shown in green if detected as a TIS by mass spectrometry in >1 biological replicate. Mutations made to *rpoB* (2) at these sites are indicated. (**B**) Total lysates of *Msm* carrying wild-type (WT) *rpoB*, *rpoB*(2), or mutants of *rpoB*(2) were analyzed by immunoblot with GroEL as a loading control. The arrows indicate bands that showed reproducible changes in intensity in *rpoB*(2) 535 (red arrow) or 742 (blue arrow). The yellow asterisk indicates a non-specific FLAG cross-reacting band in *Msm* total lysates. Data shown are from a single anti-FLAG or anti-GroEL image with lanes reordered for clarity. Data are representative of three independent experiments. (**C**) Integrated band intensities were normalized to total integrated lane intensity for bands indicated in (**B**) (see Fig. S3 for analysis of all reproducibly detected bands). Data are averages ± S.D. from *n* = 3 independent experiments. One-way ANOVA with Dunnett’s multiple comparison analysis was performed to determine the significance of each mutant compared to *rpoB*(2) (*P < 0.05; **P < 0.01).

To confirm that the TIS mapped by mass spectrometry drive the production of smaller proteins, we attempted to abrogate initiation by introducing silent mutations in the start codons of several observed TIS (Fig. 6A). We chose two TIS that were detected only in *rpoB*(2) and *rpoB*(3): codons 742 (CTG in both variants) and 797 (TTG and CTG, respectively). In addition, codon 535 is a TIS (GTG) that was detected in all *rpoB* genes. We refer to these mutants by the residue position of the detected TIS, that is, 535, 742, and 797. Each change was expected to affect the expression of a single protein isoform. Compared to expression from *rpoB*(2), mutants 535 and 742 each displayed by immunoblot a significant decrease in a single band while no reproducible changes were observed in other bands or from mutant 797 (Fig. 6B and C; Fig. S3).

Although the TIS at residue 535 was detected by mass spectrometry for all *rpoB* genes (Fig. 6A), the band that is altered in the corresponding TIS mutant of *rpoB*(2) was not detected by immunoblot for WT (Fig. 6B). Assuming that the product initiating from residue 535 is below the threshold of immunoblot detection for the WT, why would expression from *rpoB*(2) increase when this sequence segment is the same as in WT (Fig. 6A)? We hypothesized that the level of full-length transcript is higher for *rpoB*(2) than for WT, perhaps because increased translational activity on the recoded transcript further stabilizes the message. Indeed, additional qPCR on *rpoB*(2) showed that the amount of full-length transcript (amplicon A in Fig. 4) produced from *rpoB*(2) is higher than from the WT (3.1-fold change, 95% confidence interval: 1.4–6.7).

Based on a fit to the migration standards, the estimated molecular weights of the affected bands were 51 and 39 kDa, whereas the predicted mass of the products from the corresponding TIS were 73 and 44 kDa. The altered bands are both smaller than expected, suggesting a systematic error in sizing. This could be due to an inherent limitation in the resolving power of PAGE and/or anomalous migration of truncated RpoB products. Nevertheless, the relative size of the affected band between the two mutants and reproducible alteration in a single band in each mutant suggest that the sequence changes had the expected impact and that codons 535 and 742 correspond to bona fide intragenic TIS. These data also support the overall validity of the intragenic TIS mapped by mass spectrometry.

### Transcription initiation motifs are embedded in rare codon pairs

**Fig 7:**
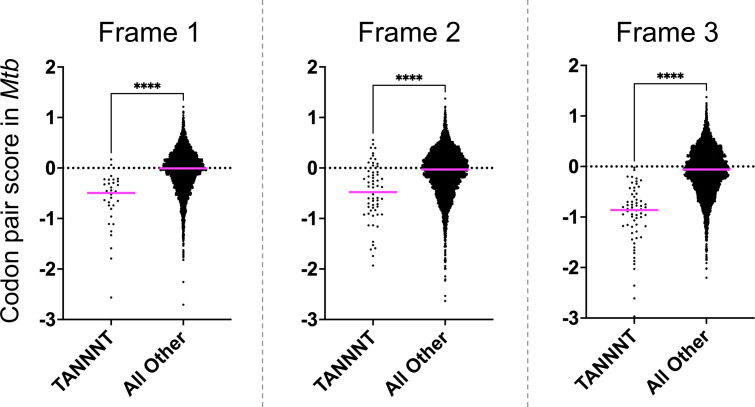
The −10 consensus sequence TANNNT is depleted in all three frames across the *Mtb* coding genome. Codon pair scores were calculated for each frame (in-frame, frame+1, frame+2) in the *Mtb* coding genome. The scores for codon pairs encoding TANNNT were compared to all others. Data and averages are shown. An unpaired *t*-test was performed to determine statistical significance (****P < 0.0001).

Because gene codon pair score does not correlate with the number of proteins produced, we hypothesized that recoding causes intragenic transcription initiation because particular rare codon pairs encode TSS motifs. To test this hypothesis, we selected codon pairs containing TANNNT, which is the −10 element most frequently observed at endogenous TSS in both *Mtb* and *Msm* (28, 29). Codon pairs encoding TANNNT were indeed significantly rarer than all other codon pairs (Fig. 7). Since transcription is a frame-independent phenomenon, the second and third reading frames of a coding sequence should also show selection against −10 elements. We, therefore, calculated the codon pair scores in frames 2 and 3 (strictly speaking, these are triplet pair scores calculated for hexamers, since these frames are generally not translated and thus the term codon does not apply). As predicted, hexamers including TANNNT were also depleted in frames 2 and 3 (Fig. 7).

## DISCUSSION

We set out to attenuate protein expression by increasing the frequency of rare codon pairs in *Mtb* genes by synonymous mutation. Instead, we found that synonymous gene recoding yielded promiscuous intragenic transcription initiation. The resulting mRNAs, in turn, yielded stable protein products with diverse and highly reproducible sizes and yields. These phenomena were also observed when synonymous recoding was random rather than explicitly designed to introduce more rare codons.

We found that recoding does not consistently attenuate full-length protein expression in mycobacteria (Fig. 2E through G). This is contrary to our original hypothesis and to previous publications in codon pair deoptimization that reported the attenuation of fitness in viruses and gene expression in yeast (6, 7). The effects of modulating codon pair bias—and making synonymous mutations in general—have not been systematically and broadly examined in bacteria. Overall, the relationship between codon pair bias score and gene expression on the RNA and protein levels needs a detailed examination. Indeed, optimizing codon pair bias has yielded conflicting reports in viruses: Optimization in one case increased, but in another case decreased, protein expression (6, 32). The lack of a consistent correlation between gene expression and codon pair bias on the gene (or genome) level is compatible with our conclusion that changes in expression depend instead on the introduction of specific codon pairs.

To our knowledge, this is one of the first reports demonstrating that synonymous gene recoding can give rise to intragenic gene expression in any organism. Our work clearly demonstrates that synonymous mutations can affect not only how proteins are translated but how mRNA is transcribed. This finding undermines the long-standing paradigm that synonymous mutations are silent. The idea that synonymous mutations can cause phenotypes is not new (1, 33 – 37). Others have established that synonymous mutations recoding single codon usage can affect mRNA folding and accessibility (36), longevity (38), and rates of translation (39, 40). Also, rare codon pairs may attenuate genes in part by causing inefficient translation elongation (6 – 10). More specifically, some codon pairs cause structural changes in the mRNA that slow down translation by stalling the ribosome at those junctures (8). Other studies have implicated post-transcriptional deficits such as decreased mRNA stability (9, 41). Prior research in codon pair deoptimization has been performed predominantly in eukaryotic systems, which might lead to speculation that intragenic protein expression is a mycobacteria-specific phenomenon. However, the dearth of data supporting the expression of smaller proteins could plausibly result from conventions in reporting and detection using native antibodies with unknown epitopes (vs our use of a C-terminal-specific epitope).

Here we found that synonymously recoding genes gave rise to intragenic, functional TSS and posit two explanations for this outcome. First, recoded sequences may boost initiation at endogenous iTSS that are largely conserved between wild-type and recoded regions but which are not observed in the wild type because *cis* elements elsewhere within the gene suppress or prevent their activity (i.e., they are cryptic). In this scenario, recoding could release these constraints and lead to the detection of additional iTSS. Overall, iTSS are not unprecedented in mycobacteria as transcriptome-wide TSS mapping studies have aligned iTSS to two of the genes selected for this study, *rpoB* and *ndh. Mtb rpoB* (*Rv0667*) may contain up to 10 different iTSS in addition to 2 TSS in the promoter region that can account for full-length protein (27). The *Mtb ndh* gene is also reported to contain one iTSS, as identified by two different studies (27, 28); none have yet been identified iTSS in *mmpL3*. The prevalence of iTSS in endogenous genes supports the possibility that, rather than forming new transcription sites, synonymous recoding is allowing or augmenting transcription initiation at existing cryptic iTSS.

Alternatively, recoding may create entirely new TSS, for example, by introducing new intragenic promoter elements. As with other species, *Mtb* and *Msm* lack a −35 consensus sequence and may therefore initiate transcription with just a −10 motif (27 – 29). The particulars of mycobacterial translation may render the mycobacterial genome permissive not only to new TSS but also transcripts that are then able to be translated. Mycobacteria readily translate transcripts that begin with just a start codon: As much as a quarter of mycobacterial mRNAs are leaderless and lack either a 5′ untranslated region or SD motif (27 – 29). As we noted above, iTSS are commonly found in mycobacteria, indicating that mechanisms already exist to allow intragenic expression. While our study was in progress, Rodriguez et al. showed in *E. coli* that synonymous recoding of a marker gene led to a new iTSS and boosted expression from an existing TIS in the antisense direction (42). These observations suggest that promoters readily arise in bacterial genomes and that even less extensive synonymous mutations than the ones we made here are sufficient to create functional promoters. Indeed, up to 10% of randomly generated 100-nt sequences are likely to contain a functional promoter in *E. coli* (43).

Moreover, the relative promiscuity of transcription initiation machinery poses the question as to why iTSS are not more common in endogenous open reading frames. Although our study design did not allow us to catalog all rare codon pairs that can result in iTSS, we confirmed that synonymously recoding genes to contain more rare codon pairs led to aberrant gene expression. Focusing on known transcription initiation motifs, we showed that codon pairs containing TSS motif “TANNNT” are rare throughout the *Mtb* coding genome and in all three frames. Taken together, these data not only offer an explanation for how codon pair deoptimization can cause new iTSS but also suggest that codon pair biases—and, more broadly, codon context biases—have evolved to curb pervasive transcription in coding regions. We speculate that such biases may serve to help cell machinery discriminate between coding and non-coding sequences.

On the protein level, a surprising result from our data was the detection of numerous N-terminal peptides that would have originated from within the ORF of wild-type *rpoB*. Evidence for intragenic TISs in *Mtb* is not unprecedented: Translation initiation is known to occur intragenically in mycobacteria and other bacteria (44). Smith et al. inferred by ribosome footprinting that intragenic translation initiation is pervasive in *Mtb* and predicted four TISs in endogenous *rpoB* that mapped to RpoB residues 154, 534, 718, and 867 (45). Our results provide direct evidence on the protein level for one of these sites via the detection of an N-terminus at residue 534. However, we also detected an additional 12 TIS resulting from wild-type *rpoB* that were not inferred by Smith et al.; 10 of these were confirmed by detection from *rpoB*(2) or *rpoB*(3) samples. The difference in detected TIS in our work vs Smith et al. may indicate the differences in sensitivity between methods used to report TIS and supports the use of multiple approaches to study these sites.

Ultimately, does the expression of smaller proteins from intragenic start sites have demonstrable biological consequences? While answering this question is beyond the scope of the current study, we note that the isoforms are probably not toxic since we did not observe any obvious changes in bacterial growth for strains containing recoded variants relative to empty vectors or wild-type controls. Since all strains used in this study contained the endogenous copy of the recoded gene, we do not know whether the smaller isoforms can modulate the function of the full-length protein. It is tempting to speculate that, independent of physiological function in bacteria, smaller protein isoforms could enhance antigen presentation by the host and provoke an enhanced specific immune response.

Our discovery that synonymous mutations can trigger new transcription may inform best practices in genome annotation, gene engineering, and recombinant genetics. A precise understanding of how nucleotide sequences encode cues for gene expression is critical for accurate genome annotation. Next-generation sequencing technologies continue to rapidly expand genome repositories, but the utility of these data is predicated on the accurate identification of promoters and open reading frames. We provide evidence that bacterial promoters readily arise within open reading frames from synonymous mutations, and this lowers the previously held threshold for transcription initiation (and translation initiation) in agreement with a growing body of research showing that transcription initiation is a relatively promiscuous process in mycobacteria. In addition, codon context optimization has been reported to impact protein production to a greater extent than codon usage optimization alone in both prokaryotes and eukaryotes (46 – 48). Understanding the relationship between codon pairs and codon context more generally is thus necessary to improve recombinant protein research.

Synonymous mutations are broadly accepted as benign and, by that logic, have been categorically excluded in drug resistance screens and studies. A recent report on synonymous mutations in drug-resistant *Mtb* showed that, although less common than non-synonymous drug resistance mutations, a number of synonymous mutations are associated with drug resistance phenotypes (49). Of note, others have recently demonstrated that synonymous mutations are non-neutral: In a yeast study that characterized 450 mutated variants of 21 genes, synonymously mutating 150 nt-segments tended to confer fitness deficits in 16 of the 21 genes tested (50). Although the exact mechanisms for the non-neutrality of those synonymous mutations in yeast are yet unclear, we have begun addressing here the mechanisms of the unintended consequences for synonymous mutations. Delving further into the mechanisms by which synonymous mutations can disrupt normal gene expression may lend greater insights into how bacteria may use these so-called silent mutations to alter the phenotype and perhaps even develop drug resistance.

This report is the first to offer mechanistic insight into the attenuation of bacterial pathogens by codon pair deoptimization. In doing so, our results inform the implementation of dinucleotide and codon biases to develop live-attenuated vaccines. Especially given the rise of drug-resistant bacteria, vaccine development is an increasingly vital strategy in combating bacterial infectious diseases. Although full-length protein expression was not consistently attenuated, synonymous recoding of essential genes may nevertheless impact mycobacterial survival, pathogenicity, and immunogenicity. Further studies of the biological mechanisms by which codon pair preferences influence bacterial transcription, translation, and physiology will inform the application of this intriguing vaccine strategy.

## MATERIALS AND METHODS

### Bacterial strains and culture conditions

Experiments were performed in *M. smegmatis* (*Msm*) mc^2^155 (ATCC 700084) or *M. tuberculosis* (*Mtb*) mc^2^6020 (H37Rv Δ*lysA* Δ*panCD*; gift of William Jacobs) (Table S1) (51). *Msm* was grown in Middlebrook 7H9 media (HiMedia) supplemented with 1% (wt/vol) casamino acid (BD), 0.2% (vol/vol) glycerol, 0.2% (wt/vol) glucose, 0.05% (vol/vol) Tween80 or 7H11 agar (HiMedia) supplemented with 10% (vol/vol) oleic acid/dextrose/catalase supplement (OADC), 0.5% (vol/vol) glycerol, and 0.05% (vol/vol) Tween80. *Mtb* was grown in Middlebrook 7H9 supplemented with 10% (vol/vol) OADC (BD), 0.2% (vol/vol) glycerol, 24 mg/L pantothenate, 80 mg/L L-lysine, and 0.025% (vol/vol) Tyloxapol or 7H11 agar supplemented with 10% (vol/vol) OADC, 0.5% (vol/vol) glycerol, 0.2% (wt/vol) casamino acid, 80 mg/L lysine, and 24 mg/L pantothenate. NEB5-alpha Competent *E. coli* (C2987, NEB) was used to propagate plasmids and was cultured in LB medium or grown on LB agar. All liquid cultures were grown at 37°C with shaking at 250 rpm for *Msm* and 110 rpm for *Mtb*. Strains containing selectable markers were cultured on a medium with 50 µg/mL of hygromycin (Hygromycin B Solution, Mirus) or 25 µg/mL of kanamycin (GoldBio) for mycobacterial strains and 100 µg/mL of hygromycin or 50 µg/mL of kanamycin for *E. coli*. See Table S2 for a list of key reagents.

### Calculation of codon pair scores

The complete H37Rv genome was collected (per Mycobrowser 3/23/2021 release, acquired 6/30/2021) for processing; non-coding and RNA-encoding genes were excluded from the analysis (52). First, the number of times any codon pair occurred in-frame in all coding sequences was recorded as the observed frequency of each codon pair. Next, the expected frequency was determined using an algorithm that controlled for codon usage as well as the distribution of rare and common codons across coding genes. The algorithm randomly shuffled synonymous codons within each gene to generate 10,000 synonymous random-codon-shuffled sequences. The number of times a codon pair was observed in a random-shuffle sequence was then averaged across all 5,000 iterations (sum of all codon pair frequencies/number of iterations). The number of occurrences of a codon pair in a given random-codon-shuffle was then totaled across the entire coding genome and averaged by the total number of iterations (i.e., 10,000). This value (the average number of times a codon pair occurs across 5,000 randomly shuffled sequences) is an approximation for the expected frequency of a given codon pair. A CPS was calculated for each codon pair by the following formula: $CPS=ln\left(\;\frac{observed\;frequency}{expected\;frequency}\;\right)$ .

### Generation of recoded variants

Codon pair deoptimized genes were designed by synonymously recoding a length of *Mtb rpoB* (gene locus Rv0667), *mmpL3* (Rv0206c), or *ndh* (Rv1854c) to maximize the number of deoptimized codon pairs. Recoded regions were codon pair deoptimized in a similar manner to previously published methods (53). Briefly, codon pairs were shuffled synonymously at random one million times within each recoded segment of *rpoB*, *mmpL3*, or *ndh*. Shuffles that increased the number of rare codon pairs were selected to generate deoptimized sequences. For the *rpoB* deoptimization score variants (range of CPB scores) used in the score variation study, deoptimized sequences were selected that matched a designated range of CPB scores (= 0, 0.01, 0.02, or −0.05). For the maximally deoptimized experimental variants (*rpoB*(1), (2), (3); *mmpL3*(1), (2), (3); *ndh*(1), (2)), recoded codons were reverted to the wild type at positions where the original codon offered a comparable or higher codon pair score.

### Molecular cloning

For a list of all vectors and primer sequences, see Tables S3 and S4. Wild-type genes *rpoB*, *ndh*, and *mmpL3* were amplified from *Mtb* H37Rv genomic DNA by PCR and inserted into pGW1-6C (gift of Tom Alber, University of California, Berkeley) by InFusion (Takara Bio) for wild-type genes or by ligation for recoded gene variants using restriction sites SpeI and NdhI. Recoded gene variants were obtained by synthesis and cloned into pUC57 by GenScript Biotech. Wild-type and recoded genes were also subcloned from pGW1-6C into the integrating vector pMV306 and episomal pMV261 (25) via the XbaI and ClaI restriction sites using ligation or InFusion. All restriction enzymes and T4 ligase were purchased from New England Biosciences.

The *rpoB* codon pair score variants with CPBs of 0, 0.01, 0.02, or −0.05 were synthesized in ~1.2 kb fragments (Twist Bioscience) and assembled by PCR into a final product of ~3.6 kb each. Variants were subsequently cloned into pGW1-6C by Gibson Assembly (NEB). Nonsense mutations were generated by site-directed mutagenesis using primers onk046 and onk047 to change the *rpoB* start codon TTG to stop codon TAG. Deletion mutants for *mmpL3* and *ndh* were similarly cloned using site-directed mutagenesis primers designed to delete the first 60 nucleotides of the open reading frame. Transcription terminator constructs were cloned from the pGW1-6C constructs by restriction digest with XbaI and SpeI to first remove the promoter. The plasmid was re-circularized using primers omlp759 and omlp760 to insert the *E. coli rrnB* T1 terminator or omlp761 and omlp762 to insert a T2 terminator (26). The *rpoB* synonymous mutant variants with point mutations at codons corresponding to residues 535, 742, and 797 were generated by site-directed mutagenesis using primers onkh083 and onkh084 to substitute nucleotide 1605g > c; primers onkh085 and onkh086 were used to substitute nucleotide 2226g > c; primers onkh087 and onkh088 were used to substitute nucleotide 2386t > c.

### Generation of bacterial lysates

*Msm* was grown in 10 mL cultures for immunoblotting or 100 mL cultures for affinity purification to an optical density at 600nm (OD_600_) of 1-1.5. Cells were pelleted by centrifugation at 3,000 *g* for 10 min and washed in sterile phosphate buffered saline (PBS) or Tris-HCl buffered saline (TBS) as noted further below. Decanted pellets were stored at −80°C and thawed just prior to lysis under buffer conditions described in further detail in the following sections. After thawing on ice, cells were resuspended in buffer and lysed with 0.1 mm zirconia beads (BioSpec) by bead beating (Beadruptor, Omni International) for 30 s at 6 m/s followed by 5 min incubations on ice for a total of 3 cycles. Lysates were cleared by centrifugation at 5,000× *g* and protein concentration was quantified by Pierce BCA Protein Assay (Thermo Fisher).

### Immunoblotting

Thawed and washed cell pellets were lysed in 1 mL sterile PBS with 10% (vol/vol) glycerol and 1× cOmplete mini protease inhibitor cocktail (Roche). Ten micrograms of protein were separated by SDS-PAGE and transferred to a nitrocellulose membrane (Bio-Rad). Membranes were probed with α-FLAG M2 (Sigma-Aldrich F1804, 1:1,000 dilution) or α-GroEL (Santa Cruz #5177, 1:5,000), followed by IRDye 800CW Goat anti-Mouse IgG secondary antibody (LI-COR, 1:15,000) and imaged (Odyssey Cx scanner, LI-COR). Images were analyzed for integrated intensities by ImageJ (54) and molecular weight estimation by GelAnalyzer 19.1 (www.gelanalyzer.com) (55).

### RNA isolation

*Msm* was grown in 10 mL cultures overnight to OD_600_ of 1–1.5. Cells were pelleted by centrifugation, resuspended in 1 mL of TRIzol LS (Invitrogen), and stored at −20°C. TRIzol resuspensions were thawed on ice before lysis by bead beating (Beadruptor, Omni International) for 30 s at 6 m/s followed by 5 min on ice for a total of 3 cycles. Total RNA was extracted from the aqueous phase by isopropanol precipitation. RNA prepared for qPCR was then purified using columns according to the manufacturer’s instructions (Qiagen RNeasy kit). Precipitated RNA for 5′ RACE or column purified RNA for qPCR was then treated with DNase using a commercial kit (Turbo DNAse, Ambion, AM2238). Total RNA yield was verified on a 2% agarose gel by visual inspection. To confirm the removal of DNA, DNase-treated samples were used as templates for qPCR targeting 16S rRNA using primers onj005 and onj006, and sample purity was checked by comparing absorbance at 260 vs 280 nm (ND-1000, Thermo Fisher).

### qRT-PCR analysis of terminator-driven *rpoB* variants

RNA samples were used as templates for cDNA synthesis with random primers (Verso Kit, Thermo Scientific). Primer efficiencies were validated by standard curves of amplication from the plasmid templates pNKH008, pNKH009, or pNKH010 (Table S3) The cDNA libraries were diluted 10-fold and 2 µL of the dilution were used for qRT-PCR analysis (Power SYBR Green PCR Master Mix, Applied Biosystems) on a LightCycler 480 Instrument (Roche). Ct values were calculated by LightCycler 480 Software (Roche) and normalized using 16S rRNA as a housekeeping gene. Primer efficiencies were verified by standard curves. Gene expression fold change was calculated using the delta-delta Ct method (Livak and Schmittgen, 2001).

### Construction of 5′-end-mapping libraries

5′-End-mapping libraries were constructed using a protocol similar to those previously published by others (29, 56, 57). RNA was isolated from *Msm* expressing 5′ transcription terminator *rpoB* as described above. DNase-treated RNA was ligated with a 5′-hydroxylated “Processed Start Site” (PSS) RNA adaptor oligomers (onkh031) by RNA Ligase 1 (Promega or NEB) and then purified to remove excess oligomers (NEB Monarch RNA Cleanup kit). RNA was then treated with RNA pyrophosphatase (NEB) and then ligated with a “TSS” RNA adaptor (onkh032). PSS- and TSS-labeled RNA was then used as a template for cDNA synthesis by random primers (Verso Kit, Thermo Scientific).

For 5′ mapping, 5′ ends were amplified by tiling PCR with a TSS tag-specific forward primer (onkh037) or a PSS tag-specific forward primer (onkh175) and one of 7 gene-specific reverse primers with annealing sites spanning the length of *Mtb rpoB* wild type or variants at ~500 bp intervals (Table S4) for a total of 14 reactions per template. PCR products were pooled by template and purified (Nucleospin, Takara). RACE amplicons were end repaired, A-tailed, and an Illumina-compatible barcode was ligated using the Kapa HyperPlus DNA Library Preparation kit (Roche). Individually barcoded libraries were amplified with Kapa HiFi HotStart DNA Polymerase (Roche) and quantified with Qubit dsDNA High Sensitivity Assay (Invitrogen). Library quality was validated with Agilent Bioanalyzer to determine the appropriate bp size. Libraries were pooled and quantified by qPCR with Kapa Illumina/Universal Library Quantification Kit (Roche). The pooled library was then sequenced on an Illumina MiSeq (PE150 with index read).

### Sequencing analysis

Sequence files (FASTQ) were trimmed for Illumina universal adapters with Cutadapt v4.0 (58). Quality control was done using FastQC v0.11.8 (59). Reads were selected for 16 base start sites (TSS or PSS) at the beginning of read 1 with 2 mismatches allowed using SeqKit v2.2.0 (60). We removed the start site sequences from the first 16 bases and aligned trimmed sequences to the rpoB gene (wild-type and three variants) using STAR v2.7.8a with default parameters (61). Unique alignments were selected by SAMtools v1.14 (62). Downstream statistical analysis was performed using basic functions and modules from Python v3.8.8, pandas v1.2.4 (63, 64), and Microsoft Excel.

We compiled all sequences preceded by a start tag (TSS or PSS) that also aligned to one of our genes of interest as follows (65): First, we normalized the TSS hit list against the list of PSS-tagged 5′ ends, retaining only the ends that had were 10-fold or more abundantly TSS-tagged vs PSS-tagged. 5′ Ends tagged with predominantly PSS sequences were eliminated from our hit list as processed starts. Of the remaining hits, we further selected only 5′ ends with the highest coverage in a 10 nt window to eliminate any redundant start sites resulting from imprecise transcription initiation (65). This was done prior to inferring the transcription start site vs processed sites from the 5′-end of aligned sequences.

### Protein affinity enrichment

Thawed and washed cell pellets were lysed in 3 mL sterile TBS with 10% glycerol, 1% Triton X-100, and a protease inhibitor cocktail (Roche). After protein quantification, whole cell lysates were precleared as follows: Lysates were incubated on a rotator at room temperature for 1 h with 7 µg/mL final concentration Immunoglobulin G1 (Sigma-Aldrich M5284) and then affinity purified with Protein G Magnetic Beads (Pierce). Briefly, beads were equilibrated according to the manufacturer’s instructions. 1/100th lysate volume of resin was added to the lysate/IgG mixture and incubated at room temperature for 1 h with gentle mixing. After the beads were collected with a magnetic separator, the supernatant was removed and used as precleared input for affinity purification with 1/30th volume α-FLAG M2 Magnetic Beads (Millipore M8823). Beads and supernatant were incubated on a rotator overnight at 4°C. The resulting supernatant was collected and the beads were washed three times with 20× resin volume TBS. Elution buffer was prepared immediately before use by adding 3XFLAG peptide (Sigma F3290) to TBS to a final concentration of 150 ng/µL. Proteins were eluted by incubating with 2× resin elution buffer for 15 min at 22°C with rotation. This elution step was repeated and eluates were pooled. The final output was validated by silver stain (Pierce Silver Stain Kit, Thermo Fisher) and visual inspection prior to processing for mass spectrometry.

Eluates were reduced, alkylated, and separated on SDS-PAGE gel. All protein bands in each sample were pooled by excising the entire gel lane, followed by N-terminal labeling with triethylammonium bicarbonate and trypsin in-gel digestion. An aliquot of the resulting desalted peptides was analyzed with an EASY-nLC 1200 coupled to a Thermo Fisher Q Exactive HF-X Mass Spectrometer operated in data-dependent mode. The resulting spectral data were searched against *M. tuberculosis* RpoB wild-type and recoded sequences using Byonic and N-terminally modified peptides were verified manually.

### Inferring start codons from N-terminal labeled MS/MS Hits

We compiled all spectra showing an N-terminal diMe, Ac, or fMet. We excluded any spectra falling below a threshold Byonic score of 300, which measures the quality of a given peptide-spectrum match (66). Any diMe or Ac residues immediately following an R or K tryptic cut site were excluded to avoid false positives. All hits indicating diMe-M or Ac-M were also excluded given that any N-terminal Met would be expected to be fMet. All hits indicating diMe-K were excluded because of possible side-chain modification. In processing the Ac hits, all hits that followed a K or R residue were excluded. Despite evidence that Ac-T and Ac-S are frequently found at the N-terminus, we also excluded those amino acids because of possible side-chain modifications, as well as Ac-K, Ac-Y, and Ac-H (67 – 69). Finally, N-terminal residues were inferred to represent a true translation initiation event if the peptide position mapped to a codon +1 from a start codon. For this study, we accepted ATG, GTG, and TTG as typical start codons, as well as the less common start CTG (70, 71).

## SUPPLEMENTAL MATERIAL

Supplemental Figures mbio.00841-23-s0001.pdf Figures S1-S3.

Tables S1 to S4 mbio.00841-23-s0002.xlsx Strains, reagents, plasmids, and primers used in this study.

Table S5 mbio.00841-23-s0003.xlsx Transcriptional start sites detected by 5′ RACE-seq.

ASM does not own the copyrights to Supplemental Material that may be linked to, or accessed through, an article. The authors have granted ASM a non-exclusive, world-wide license to publish the Supplemental Material files. Please contact the corresponding author directly for reuse.
