# Supplementary material for: Gene recoding by synonymous mutations creates promiscuous intragenic transcription initiation in mycobacteria
Source: mBio. 2023 Oct 3;14(5):e00841-23. doi: 10.1128/mbio.00841-23 (PMC10653884; doi:10.1128/mbio.00841-23)
Supplement: Supplemental Figures — Figures S1-S3. [file mbio.00841-23-s0001.pdf]

Gene recoding by synonymous mutations creates promiscuous intragenic transcription initiation in mycobacteria

Nuri K. Hegelmeyer[a], Lia A. Parkin[b], Mary L. Previti[a], Joshua Andrade[c*], Raditya Utama[d], Richard J. Sejour[b], Justin Gardin[b*], Stephanie Muller[d], Steven Ketchum[b], Alisa Yurovsky[b], Bruce Futcher[b], Sara Goodwin[d] Beatrix Ueberheide[c,e], Jessica C. Seeliger[a#]

[a] Department of Pharmacological Sciences, Stony Brook University, Stony Brook, New York, USA

[b] Department of Microbiology and Immunology, Stony Brook University, Stony Brook, New York, USA

[c] Proteomics Laboratory, New York University Grossman School of Medicine, New York, New York, USA

[d] Cold Spring Harbor Laboratory, Cold Spring Harbor, New York, USA

[e] Department of Biochemistry and Molecular Pharmacology, New York University Grossman School of Medicine, New York, New York, USA.

Supplemental Information

- **Figure S1.** Expression of smaller proteins from recoded genes are not an artifact of gene copy number or selection with the translational inhibitor hygromycin
- **Figure S2.** Substituting a TAG stop codon for the start codon does not abolish expression of full-length Ndh or MmpL3

- **Figure S3.** TIS mutants of rpoB(2) display changes in the levels of specific protein isoforms.

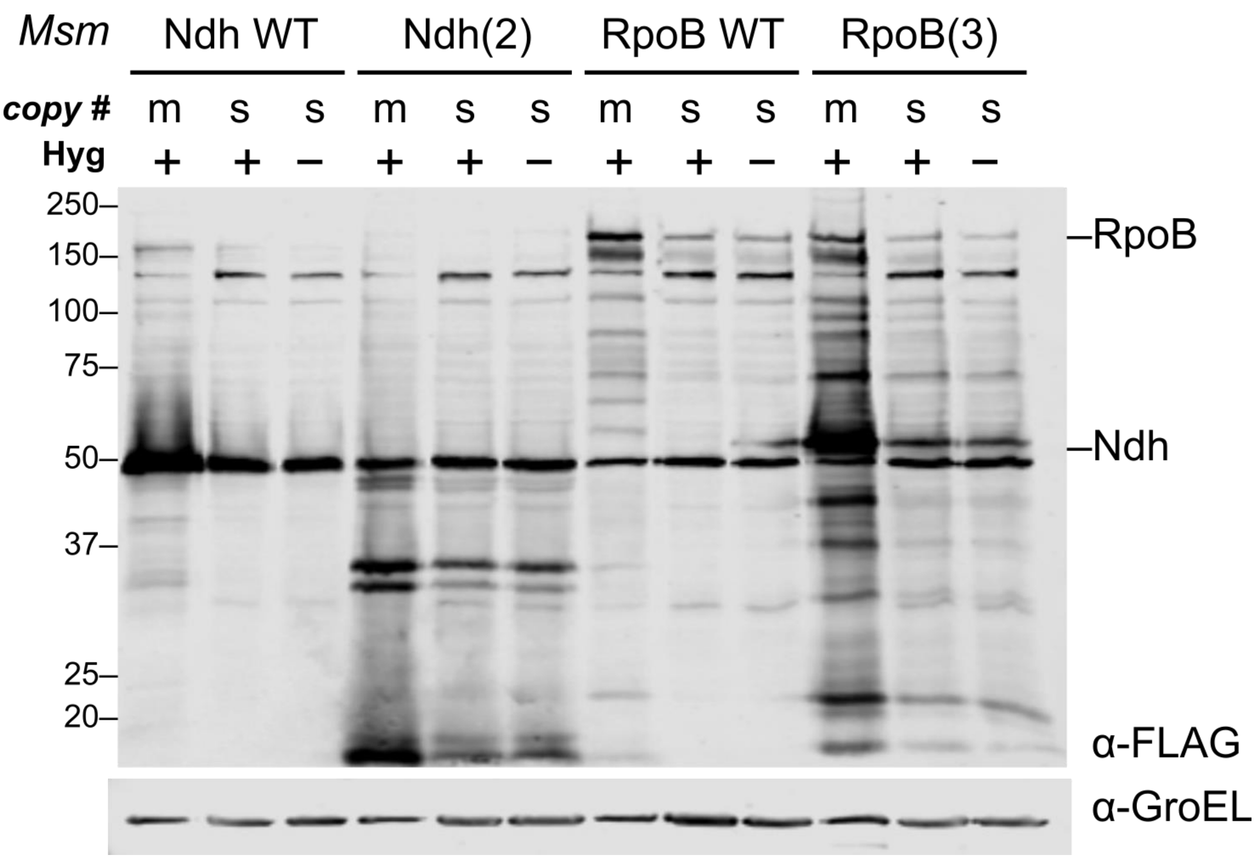

Supplemental Figure S1. Expression of smaller proteins from recoded genes are not an artifact of gene copy number or selection with the translational inhibitor hygromycin. Immunoblot of wild-type and recoded Mtb genes expressed in Msm multicopy episomal ("m") or single copy integrated ("s") plasmids. Strains with stably integrated plasmids ("s") were grown with or without hygromycin, as indicated. Blot is representative of 3 biological replicates. GroEL blot is included as a loading control.

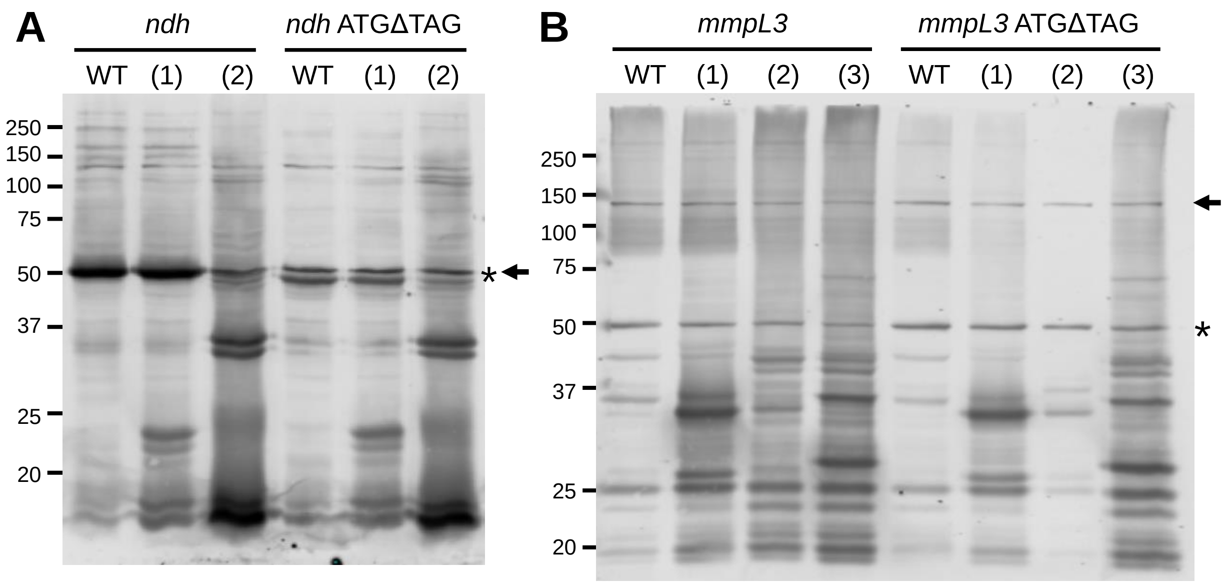

Supplemental Figure S2. Replacing the start codon with the TAG stop codon does not abolish expression of full-length Ndh or MmpL3. Anti-FLAG immunoblots of total lysates from *Msm* expressing the designated wild-type or recoded (A) *ndh* or (B) *mmpL3* constructs without (*left*) or with (*right*) the start codon substituted with the TAG stop codon (ATGΔTAG). Arrows designate the expected migration of full-length protein. Asterisks denotes a non-specific FLAG cross-reacting band in *Msm* total lysates. This band is nearly coincident with full-length Ndh (see also Figure 2I). Immunoblots are representative of 3 (*ndh*) or 2 (*mmpL3*) independent experiments.

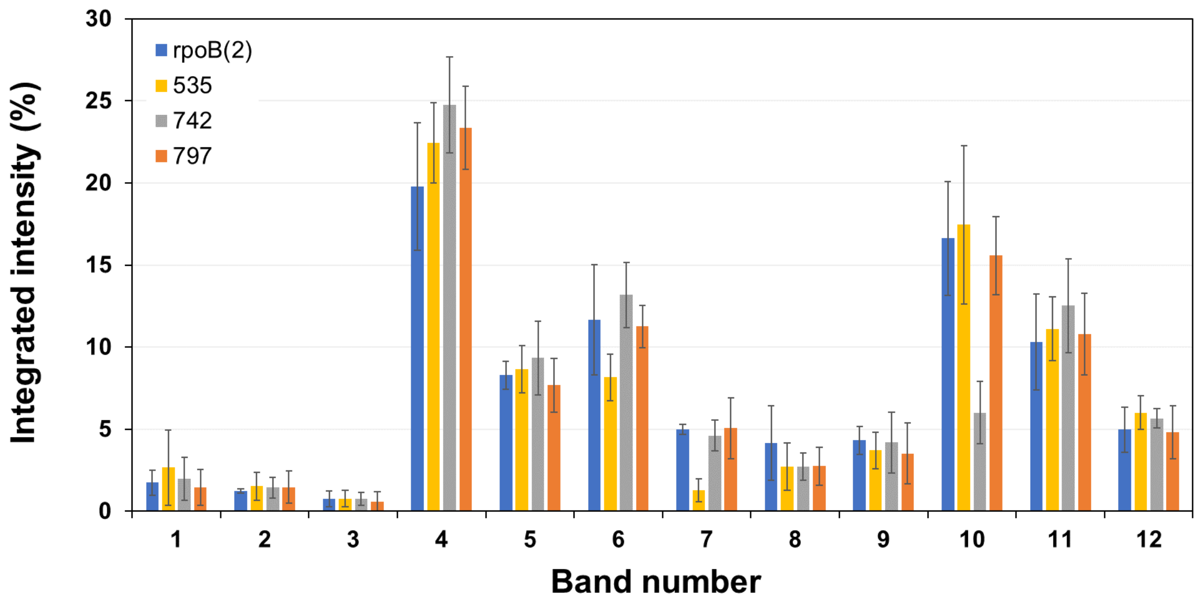

Supplemental Figure S3. TIS mutants of *rpoB*(2) display changes in the levels of specific protein isoforms. Total lysates of Msm expressing *rpoB*(2) and TIS mutants at nucleotide positions 535, 742, 797 were analyzed by anti-FLAG immunoblot (see Figure 6B). Integrated intensities for twelve bands that were detected in all of 3 biological replicates were normalized by total integrated lane intensity for each gene variant. Data shown are the average of three biological replicates ± S.D. Data in Figure 6C are from bands 7 and 10.
